# Taxane-Producing Fungi Isolated from *Taxus globosa* Tree Bark

**DOI:** 10.3390/microorganisms13020300

**Published:** 2025-01-29

**Authors:** Jocelyn Guadalupe Guevara-Sánchez, María Guadalupe Aguilar-Uscanga, Carlos Augusto Ledesma-Escobar, Claudia Castro-Martínez, Renaud Condé, Bernardo Sachman-Ruíz, Sandra del Moral

**Affiliations:** 1 Laboratorio de Bioingeniería-UNIDA, Tecnológico Nacional de México, Instituto Tecnológico de Veracruz, Veracruz 91897, Veracruz, Mexicomaria.au@veracruz.tecnm.mx (M.G.A.-U.); 2 Departamento de Química Analítica, Campus Rabanales, Universidad de Córdoba, 14071 Cordoba, Spain; 3 Departamento de Biotecnología Agrícola, Instituto Politécnico Nacional, Centro Interdisciplinario de Investigación para el Desarrollo Integral Regional, Unidad Sinaloa, Guasave 81100, Sinaloa, Mexico; claudiacm30@hotmail.com; 4 Centro de Investigaciones Sobre Enfermedades Infecciosas, Instituto Nacional de Salud Pública, Cuernavaca 62100, Morelos, Mexico; 5 Laboratorio de Artropodología del INIFAP CENID Salud Animal e Inocuidad, Jiutepec 62550, Morelos, Mexico

**Keywords:** taxane, endophytic fungi, *Taxus globose*, baccatin III, paclitaxel

## Abstract

The taxane molecules extracted from the bark of trees from the *Taxus* genus demonstrate anticancer activity and are the main components of the drug paclitaxel. Even though a great deal of research has been carried out to produce them synthetically, this manufacturing is still dependent on *Taxus* cell culture. Furthermore, these processes are not suitable for steady taxane production. Therefore, the search for alternative sources of taxane production has generated growing interest amongst the scientific community. The use of endophytic fungi for the obtention of taxane constitutes an attractive alternative. Here, we present an analysis of the taxane production of several microorganisms through LC–QTOF MS/MS. We isolated 21 endophytic fungus strains, identified through sequencing of their internal transcribed spacer region (ITS). The phylogenetic analysis showed the presence of 11 different *Fungus* genera, namely *Aspergillus*, *Trichoderma*, *Neurospora*, *Penicillium*, *Curvularia*, *Arcopilus*, *Biscogniauxia*, *Hypoxylon*, *Sordaria*, *Xylariales*, and *Gelasinospora*. These fungi have been previously described to synthetize distinct metabolites of medical interest, hence supporting the study of their potential taxane production. Here, we report the production of taxadiene by some of these fungi, namely *Aspergillus* sp. (1.35 μg/L)*,* TgO (0.7 μg/L)*, Trichoderma harzianum* (0.13 μg/L), and *Hypoxylon* sp. (0.3 μg/L). Furthermore, we also detected the production of baccatin III, a crucial precursor component of the drug paclitaxel. This is the first report of taxane production by fungi phylogenetically related to the *Arcopilus* and *Endoxyla* genera.

## 1. Introduction

The search for cancer treatment has been an uphill battle. This is due to the particularities of each type of cancer [1]. Amongst the anticancer drugs currently used, paclitaxel is a well-known taxane compound that was approved by the FDA in 1992 [2]. This drug was originally extracted from the bark of trees from the *Taxus* genus and has proven efficacy for treating solid tumors in breast, lung, and ovary cancer [3]. To this day, the production of taxanes depends on their extraction from *Taxus* trees that are more than 60 years old, as the concentration of paclitaxel is higher in their barks. These trees produce around 4.70 × 10^−6^ mg/g/day or 0.017% dry weight of paclitaxel [4]. In the past 30 years, several other methods for synthesizing paclitaxel have been developed, but the large number of enzymes required is a strong limitation of these approaches [5]. Additionally, the purification of the enzymatic products of taxane has proven to be daunting [6]. An alternative is to culture *Taxus* calluses from explants in solid media, which can provide the biological base material for subsequent liquid cell culture. The yield in taxane production depends on the morphology and age of the callus, with the oldest callus producing the greatest amount of paclitaxel [7]. The tree strain also influences the amount recovered, with relative quantities of around 30, 5, and 10 µg/g dry weight from *T. baccata* stems, *T. brevifolia* stems, and *T. brevifolia* leaves, respectively [8].

An alternative approach to producing paclitaxel is performing semi-chemical synthesis, starting from biological intermediate products. However, the number of enzymatic reactions needed to obtain the product limits the feasibility of this method [9]. Furthermore, the process of purifying the final products renders this method very cumbersome [10]. Another strategy for producing taxanes is to use undifferentiated cells obtained from *Taxus* calluses (plant explants grown in solid media) to extract the products [11]. These cells are the starting material for cell suspension growth. The paclitaxel production of the calluses depends on their morphology and age, with the oldest usually showing a higher production. *T. globosa* calluses yield 2.8 µg/g dry weight of paclitaxel, while *T. baccata* calluses produce 1.90–8.75 µg/g of culture [12]. One of the drawbacks of callus culture is that the transfer of oxygen and nutrients is governed by the distribution of the size of the callus fragments. Also, microorganism contamination of the culture is a common feature and can even cause the loss of the line [11].

Nevertheless, the development of in vitro *Taxus* cell suspension cultures has increased paclitaxel production, with it reaching up to 4.76 mg/L per day in *T. marei* cell lines [13]. Due to the ease of distribution of oxygen and nutrients during culture, the suspension of microorganisms in liquid media offer advantages over callus cultures. However, liquid culture tends to alter the plant’s intracellular communication and produce cell aggregates and cellular variability and instability [14]. These effects result in variations in paclitaxel production, mainly due to the fact that the cells are at different developmental stages [15].

Although alternatives for producing paclitaxel, such as chemical synthesis, the use of calluses, and the development and implementation of plant tissue culture, have been encountered, there are still no stable and robust production systems.

As an alternative to plant extraction, the presence of paclitaxel has been sought out in other cells. In 1993, Stierle et al. first reported the production of paclitaxel by an endophytic fungus isolated from *T. brevifolia*: *Taxomyces andreanae* [16]. Forty genera of fungi with the capacity to produce paclitaxel and its analogs were later identified [17]. These endophytic fungi can also produce multiple secondary metabolites.

The production of metabolites in fungi has several advantages: short cultivation times (15–20 days), lower costs of the culture media when compared to that of plant tissue cultures, and, above all, a robust and stable production system with little genetic variability [4].

*Taxus globosa*, also known as Mexican yew or romerillo, is one of the species included in the genus *Taxus*, which comprises around nine species natives to the northern hemisphere of America [18]. It is found occasionally in various states of Mexico, such as Nuevo León, Tamaulipas, San Luis Potosí, Querétaro, Hidalgo, Puebla, Veracruz, Oaxaca, and Chiapas, as well as in Guatemala, Honduras, and El Salvador [19]. Like the other species in the genus Taxus, the Mexican yew is a source of paclitaxel [20]. To date, no isolation of taxane-producing endophytic fungi from *T. globosa* has been reported. Here, we isolated and identified endophytic fungi with potential taxane production present in *T. globosa* bark and analyzed its production of taxane’s anticancer precursors.

## 2. Experimental Procedure

### 2.1. Sample Collection

*T. globosa* bark samples were collected from Acajete municipality, Veracruz state, Mexico (19°31′18.8″ N 97°03′14.4″ W), from August 2021 to April 2022. The samples were collected using a sterile scalpel, stored and transported in a sterile plastic bag with a hermetic lock to the Laboratorio de Bioingeniería in order to be processed in refrigerated conditions.

#### 2.1.1. Endophytic Fungi Isolation

The bark samples were exposed to a surface disinfection process consisting of tap water wash, immersion in 70% (*v*/*v*) ethanol for 1 min and immersion in sodium hypochlorite (20% *v*/*v*) for 3 min. Subsequently, the barks were rinsed 5 times with sterile distilled water [21]. The cortex was left to dry for 20 min inside a laminar flow hood, and then the disinfected surface was removed with a sterile scalpel and the remaining tissue was cut into fragments of approximately 1 cm^2^ to be seeded by puncture in PDA medium, Sabouraud dextrose, sorghum flour (80 g/L), corn flour (80 g/L) and oat flour (60 g/L) with chloramphenicol added (600 mg/L). The sorghum, corn and oat flour culture media were formulated through the direct grinding of the cereals, which were sieved until obtaining a thin flour. The cultures were incubated at 30 °C for 7 days. After this period, we individually subcultured the morphologically different colonies until axenic cultures were obtained. For the preservation of the pure cultures obtained, spores were collected with a solution of Tween 80 (0.1% *v*/*v*) and stored at −20 °C using pure glycerol (1:1) as a cryoprotective agent.

#### 2.1.2. Microscopical Morphology Characterization

The morphological study of the pure cultures was carried out using the adhesive tape method. Under aseptic conditions, a sheet of said material was placed on the colony to be examined, pressing gently until it adhered. The microorganisms were subsequently transferred to a microscope slide previously stained with lactophenol blue and observed under the microscope with a 40× view. The observed structures were compared with the typical taxonomic keys for each genus [22,23].

#### 2.1.3. Genomic DNA Extraction

Genomic DNA was extracted from mycelium (50 mg), which was ground using a mortar and pestle. Then, 300 µL of DNAzol^®^ solution (Invitrogen, INVITROGEN CORPORATION, Carlsbad, CA, USA) was added and left to stand for 10 min. The solution was then centrifuged (spectrafuge 16M, Radnor Corporate Center, Radnor, PA, USA) for 10 min at 11,430× *g*. The supernatant was transferred to a new tube where 150 µL of cold absolute ethanol was added and incubated for 10 min at −20 °C; then, the samples were centrifuged at 13,715× *g* for 5 min. The supernatant obtained was discarded and a wash was performed by adding 500 µL of 75% (*v*/*v*) ethanol to the pellet and leaving to stand for 5 min. The pellet was centrifuged at 13,715× g for 2 min and a second wash was performed. The supernatant was discarded and the pellet was allowed to dry at room temperature. The pellet was resuspended in 50 µL of ultrapure water (milli-Q).

#### 2.1.4. Molecular Identification Through ITS

The internal transcribed spacer (ITS) fragments were amplified using the universal primers ITS1 (5′-TCCGTAGGTGAACCTGCGG-3′) and ITS4 (5′-TCCTCCGCTTATTGATATGC-3′) from the genomic DNA obtained. The PCR reaction used 100 ng of genomic DNA mixed with 0.25 µL of 5 U/µL polymerase (Go Taq DNA polymerase, PROMEGA, Madison, WI, USA), 5 µL of 5× buffer (Go Taq Flexi buffer, PROMEGA, Madison, WI, USA), 1.5 µL of MgSO_4_ solution (25 mM), 14 µL of nuclease-free water and 1 µL of the forward and reverse primer (250 nM). Amplifications were performed in a MULTIGENE thermocycler (LabNet, Corning Life Sciences, Tewksbury, MA 01876, USA) programmed with the following reaction conditions: initial denaturation at 95 °C for 2 min, followed by 30 cycles of 1 min at 95 °C, 1 min at 55 °C and 1 min at 72 °C, with a final extension period at 72 °C for 5 min. PCR products were separated by electrophoresis (100 V 400 mA for 40 min) in a 1% (*w*/*v*) agarose gel/T.B.E. 1X buffer (89 mM Tris, 89 mM boric acid, 2 mM EDTA, pH ~8.3, etidium bromide 0.5 µg/mL). The corresponding amplicon was purified using the QIAquick kit (Qiagen, Germantown, MD 20874, USA). The O.D. 260/280 ratio of the purified DNA was evaluated using Nanodrop^®^ 2000 (Nanodrop^®^, Wilmington, NC, USA).

The samples were sequenced by the chain termination method of Sanger et al. [24] at the LANGEBIO Sequencing Unit, Cinvestav in Irapuato, Guanajuato. Regions without signal from the electropherograms were eliminated using the Bioedit 7.2 software. (Informer Technologies, Inc., 6800 Altamor Drive, Los Angeles, CA, USA). Each pair of sequences was assembled in the FASTA format and a consensus sequence was obtained with the Bioedit 7.2 software, which was later analyzed with the online BLASTn program.

#### 2.1.5. Phylogenetic Analysis

Local alignment of the consensus sequences was carried out with the BLAST program (NCBI). The sequences presenting the highest percentage of identity and coverage were selected. They were aligned in the freely available online program MUSCLE, and the best evolutionary substitution was inferred using the FindModel method, which contains Akaike and LnL information criteria. Molecular phylogenetic relationships were constructed using the maximum likelihood method, the 2-parameter Kimura distance method and standard Bootstrap analysis. The tree was rooted with *Saccharomyces cerevisiae*, using the MEGAXi program.

#### 2.1.6. Identification of Taxane-Producing Fungi by LC–QTOF MS/MS

The isolated strains were grown in Mandels–Weber medium in an orbital shaking incubator at 150 rpm and 25 °C for 14 days. After this period, the biomass was separated from the culture medium by centrifugation at 4000 rpm, 20 min (Eppendorf centrifuge 5510, Eppendorf North America, Enfield, CT, USA). The biomass was left to dry in an oven (Thermo Scientific Lab-line, Waltham, MA, USA) at 55 °C for 24 h or until reaching constant weight, while the cell-free medium was filtered with sterile 0.22 μm acrodiscs. Both samples were stored at −20 °C until further use.

#### 2.1.7. Taxane Extraction

For the extraction of taxanes, 20 mg of the dry sample was mixed with 500 µL of 3% (*v*/*v*) H_2_SO_4_ and shaken at 2000 rpm and 30 °C for 30 min (Eppendorf ThermoMixer C; Eppendorf North America, Enfield, CT, USA). Glass beads were used to homogenize the samples. The extract obtained was supplemented with 10% (*v*/*v*) NH_3_ until reaching a pH of 4. Liquid–liquid extraction was performed adding 250 µL of ethyl acetate, shaking the samples at 2000 rpm for 10 min at 30 °C. Subsequently, centrifugation at 14,000 rpm and 4 °C was performed to separate the phases. The ethyl acetate phase was recovered and evaporated under vacuum at 30 °C (Eppendorf Concentrator plus; Eppendorf North America, Enfield, CT, USA) until dry. The residue was reconstituted with 50 µL of methanol containing 1 ppm of syringaldehyde used as an internal standard.

#### 2.1.8. Taxane Purification

For the chromatographic separation of metabolites, we used the Agilent 1200 series LC system (Agilent Technologies, Santa Clara, CA, USA), fitted with a Zorbax Eclipse Plus C18 chromatographic column (particle size 1.8 µm, 3.0 × 50 mm internal diameter × mm length) with a 5 mm long guard column with the same characteristics (Agilent Technologies, Santa Clara, CA, USA). The mobile A phase was composed of water and the mobile phase B of acetonitrile. The separation gradient was carried out at a constant flow rate of 0.5 mL/min with a sample injection volume of 5 µL. The gradient was the following: from 0 to 3 min, the composition of phase B increased from 60% to 100% and was maintained at this gradient for 4 more min to ensure the elution of all metabolites from the sample. The total analysis time for each sample was 7 min. After each analysis, the column was equilibrated for 4 min to restore initial conditions.

The same chromatographic device and protocol were used for the LC–MS analysis. The chromatographic system was coupled to an Agilent 6540 quadrupole tandem time-of-flight (QTOF) mass spectrometer in high-resolution mode (Agilent Technologies, Santa Clara, CA, USA), equipped with an electrospray ionization source.

#### 2.1.9. Taxane Spectrometrical Analysis

For mass spectrometry analysis, the electrospray ionization source was operated in positive mode with the following parameters: the capillary voltage was 3500 V; the skimmer, Q1, and octopole voltages were 65 V, 130 V, and 750 V, respectively. The nebulizer gas pressure was 40 psi, and a 350 °C, 10 L min^−1^ drying gas flow was used. Data were acquired in centroid mode with extended dynamic range (2 GHz). MS scanning was performed at 6 spectra per second in the *m*/*z* range of 100 to 1100, followed by activation of MS/MS for the five most intense precursor ions using collision energies of 20 eV and 40 eV at 3 spectra per second in the *m*/*z* range of 40 to 1100. An active precursor ion exclusion window of 0.5 min was programmed to avoid repeated fragmentation of the most intense precursor ions.

In order to find an ionizing agent that would allow for correct ionization of the molecules for the mass spectrometry analysis, ionizations were performed using formic acid, ammonium acetate, ammonium formate and combinations of these agents in positive and negative ionization mode. Furthermore, to obtain further information on the fragmentation pattern of the molecule (neutral mass losses, relative intensity and characteristic fragments), analyses were performed using 20 eV and 40 eV collision energies. Taxanes were found to form more stable ions in positive mode using 5 mM ammonium acetate, which enabled ionization of baccatin III, forming an ammonium adduct ([H+NH_4_]+).

## 3. Results and Discussion

### 3.1. Endophytic Fungus Strain Isolation, Identification, and Classification

Twenty-one individual strains of endophytic fungus were isolated from the *T. globosa* barks, and 48% of these fungus strains were grown in PDA media, 19% in maize/wheat media, and 33% in sorgo flour. In general, growth media formulated with natural ingredients are the most efficient for fungus culture, though no media has proven to be universal. Each strain does present specific nutrient requirements [25]. The macro physiological characteristics of the isolated strains are presented in Table 1.

**Figure 1:**
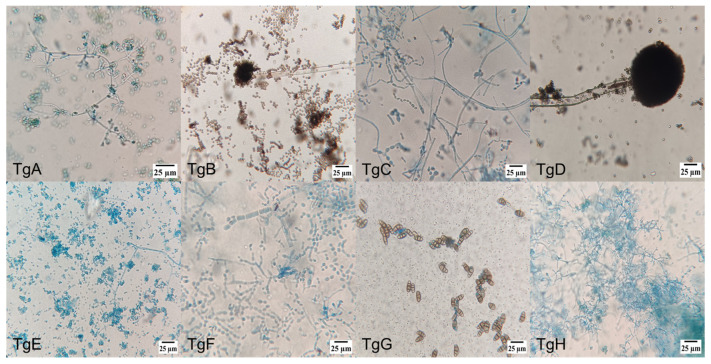

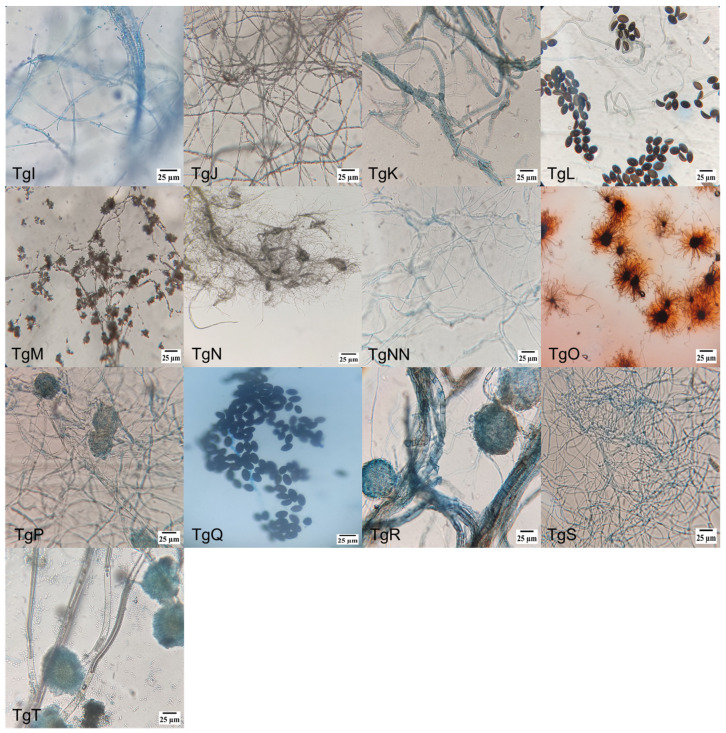
Morphology of endophytic fungi isolated from the bark of *T. globosa*: (TgA) conidiophore, phialide and conidia; (TgB) conidiophore and conidia; (TgC) conidiophore and phialide; (TgD) conidiophore; (TgE) conidiophore, phialide and conidia; (TgF) septate hyphae; (TgG) ellipsoidal conidia with septa; (TgH) conidiophore, phialide and conidia; (TgI) hyphae with microconidia; (TgJ) hyphae with microconidia; (TgK) conidiophore with denticles; (TgL) ascospores and hyphae; (TgM) hyphae with microconidia; (TgN) conidiophore and phialide; (TgNN) aseptate hyphae; (TgO) perithecium with terminal hairs; (TgP) ascospore; (TgQ) ascospore; (TgR) ascospore; (TgS) blastospore; and (TgT) conidiophore.

Figure 1 shows microscopic pictures (400×) of the different strains’ culture, where the diverse individual cell’s morphologies reinforced the identification of the endophytic mushrooms, together with molecular and taxonomic analysis. Amongst the different morphologies, conidiophores are dominant, followed by hyphae and ascospore-shaped fungus.

### 3.2. Phylogenetic Analysis of the Fungus Islolates 

Acording to the MycoBank engine and databases analysis, the isolated endophytes are members of three classes of *Ascomycota*: *Sordariomycetes* (75%), *Eurotiomycetes* (20%) and *Dothideomycetes* (5%). The strains are phylogenetically related to the genera *Aspergillus*, *Trichoderma*, *Neurospora*, *Penicillium*, *Curvularia*, *Arcopilus*, *Biscogniauxia*, *Hypoxylon*, *Sordaria*, *Xylariales,* and *Gelasinospora* (Robert, V., Stegehuis, G., & Stalpers, J. (2005). The MycoBank engine and related databases www.mycobank.org (accessed on 18 January 2023). Of the 21 isolated strains, 4 were identified to the species level through molecular analysis of their ITS region (Supplementary Material, Figure S2). These pertained to the species *Trichoderma harzianum* (TgE, TgN), *Sordaria tomentoalba* (TgP) and *Biscogniauxia mediterranea* (TgS). The rest of the isolates were identified at the genus level: the TgG isolate is phylogenetically related to the *Curvularia* clade; TgB, TgD and TgT isolates are related to the *Aspergillus* clade, while the TgC isolate pertains to the *Penicillium* clade (Supplementary Material Figure S2). 

We also show that the TgK isolate belongs to the same clade as *Xylariaceae*, *Colletotrichum* and *Sordariomycetes*. The TgJ and TgM isolates are related to the *Xylariales, Hypoxylon* and *Daldinia* clades. The TgS isolate belongs to the *Biscogniauxia* clade and the TgP, TgR, TgL, TgQ and TgF isolates to the *Sordaria* and *Neurospora* clades. The TgO samples are in the same clade as *Arcopilus, Collariella* and *Chaetomium*. TgNN relates to the *Camarops, Fusarium* and *Sordariomycetes* clades. Finally, the TgE, TgH, TgA and TgN isolates are classified as *Thricoderma* (Supplementary Material Figure S2). The fungi of the *Sordariaceae* family (including the genera *Gelasinospora, Neurospora* and *Sordaria* but excluding *Apodus* and *Diplogelasinospora*) form a monophyletic group, as previously reported [31]. The universal primers ITS1 and ITS4 used failed to amplify strain I. Species-level identification would require sequencing of the gene encoding the largest subunit of RNA polymerase II (RPB1), which has a single-copy homologous between all fungi species and has a slow rate of divergence [32].

These results are in agreement with other reports [33,34,35] that identify endophytic fungi from *T. globosa*. The distribution of endophytic fungi in *Taxus* genera is variable. Many of them, like the *Trichoderma* sp.fungi, can be found in various tree species. They are commonly used as biological control agents against phytopathogenic fungi [36]. Their secondary metabolites can also regulate the growth of the plants they colonize and activate their defense response [37]. The *Biscogniauxia* fungi is present in healthy trees as an endophyte but can become invasive under drought conditions [38]. In 2012, Ming-Jenet al. identified secondary metabolites in *Biscognauxia* with antimycobacterial capacity [39], while Silva-Hughes et al. evidenced the presence of a compound with moderate antifungal activity against the phytopathogenic fungus *Phomopsis obscurans* [40]. Also, *N. crassa* produces gibberellins, hormones that promote plant growth [41,42]. *A. terreus* and *P. citrinum* promote specific traits during plant growth, such as shoot length, shoot diameter, shoot fresh/dry weight, transpiration, stomatal conductance, photosynthesis and chlorophyll content. It also reduces the negative impacts of stem rot caused by *Sclerotium rolfsii* [43]. The *Fusarium* genus, isolated from *T. wallichiana* [44], produces a wide range of secondary metabolites that exhibit a variety of biological activities, including antimicrobial, anticancer, antiviral, antioxidant, antiparasitic, immunosuppressive, immunomodulatory and antithrombotic properties. It is also used as biocontrol against plant pathogens and nematodes, providing protection and new survival strategies for its host plants [45]. *Sordaria tomentoalba* has recently been proposed as a possible biocontrol strategy against the phytopathogenic fungus *Botrytis cinerea* [46]. Likewise, isobenzofuranones obtained from *H. anthochroum* inhibit the growth of *F. oxysporum*, *A. alternata*, *P. aphanidermatum* and *P. capsicia*. These proprieties position them as a new and useful resource for the control of phytopathogenic fungi and oomycetes of agricultural relevance [47].

### 3.3. Taxane-Producing Endophytic Fungus

**Figure 2:**
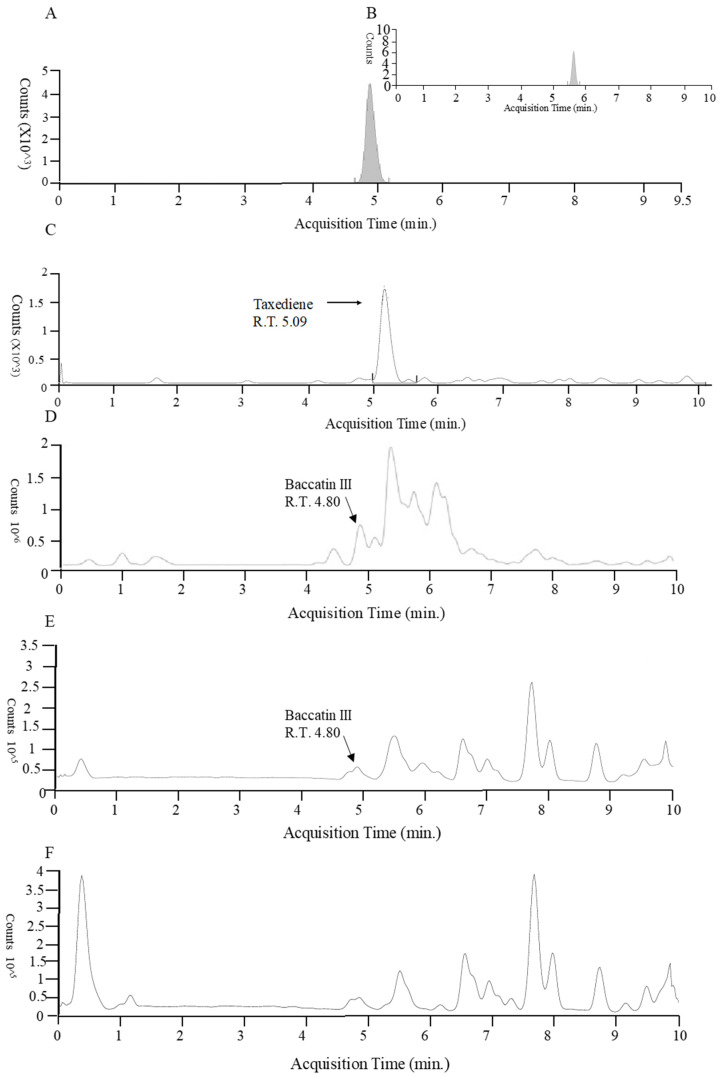
Liquid chromatography/mass spectrometry of baccatin III and taxadiene from plant cell and fungus. (**A**) Standard baccatin III. (**B**) Baccatin ammonium adduct. (**C**) Plant cell taxadiene. (**D**) *Aspergillus* sp. (TgB) Baccatin III. (**E**) (TgO) Baccatin III. (**F**) *T. harzianum* (TgE) taxadiene.

**Figure 3:**
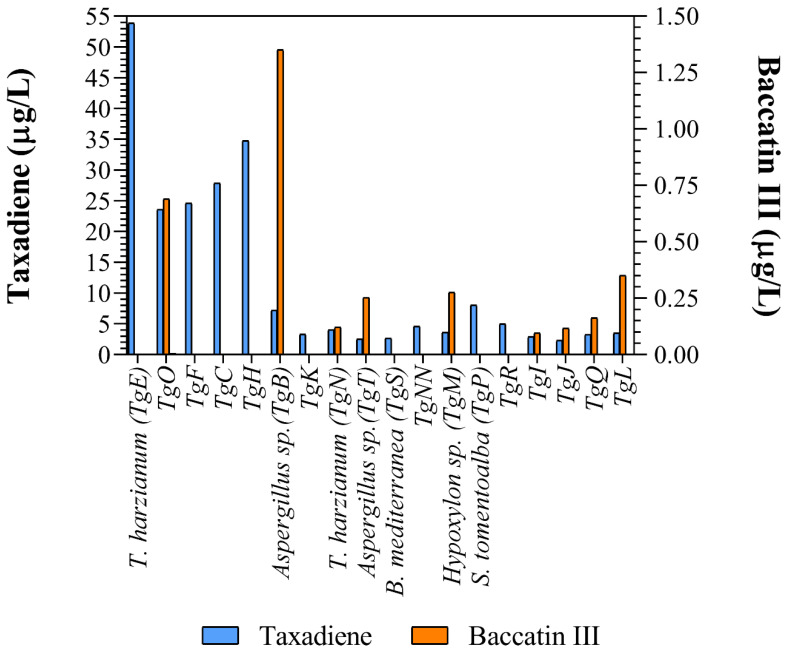
Taxane and baccatin III concentration in the supernatant of the different cultured endophytic fungi reported in this work.

Taxadiene (C_20_H_32_) and baccatin III (C_31_H_38_O_11_) were detected by liquid chromatography/mass spectrometry (Figure 2). All the identified fungus strains produced chemicals containing taxadiene ring, while only *Aspergillus* sp. (TgB), *T. harzianum* (TgN), *Hypoxylon* sp. (TgM) TgI, TgJ, TgO, TgQ and TgL produced baccatin III, the immediate precursor of paclitaxel (Figure 3).

Taxadiene ring motif production might be indicative of an alternate synthetic via since this molecule is central to the plant malevolate and metiletritol phosphate synthetic pathways. The enzymes involved in these syntheses could promote paclitaxel production in endophytic fungus [48]. Similar strains have been reported to produce higher taxane concentrations, but these differences might be due to the fungus culture conditions (Table 1). The composition of the culture media, whether solid or liquid, influences taxane production. The best media for fungus sporulation and for taxane production are M1D and MFBB, respectively. These media are rich in organic nitrogen sources, such as yeast extract and peptone [30,48,49].

Taxadiene was present in the culture media of all fungus strains, while baccatin III was detected in only nine of them. The addition of nutrient such as CuSO_4_ (0.1 ${mgL}^{-1}$), salicylic acid (10 ${mgL}^{-1})\;$ and sodium acetate (8 ${gL}^{-1}$) increased taxane production up to four times [50]. The media used in this research provides numerous salts (Ca(NO_3_)_2_, KNO_3_, KCl, ammonium tartrate, KI, FeCl_3_ and H_3_BO_3_) and amino acids (tiamin, tyrosine, biotine, pyridoxal and phenylalanine) absent from the reported media. Considering that a phenylalanine lateral chain is added to baccatin III to form paclitaxel, this addition could greatly enhance its production [51]. Furthermore, we report for the first time that fungi phylogenetically related to the genera *Arcopilus* and *Endoxyla* produce taxanes.

Fungal production of baccatin III and taxanes, in general, constitutes a biotechnological alternative to the otherwise unpractical chemical synthesis of these anticancer drugs. The fungus species found in this study are a viable substitute for their production as well as for the generation of secondary metabolites of high commercial value, such as paclitaxel. Aside from providing the precursors for these compounds, this mode of taxanes production would also avoid the overexploitation of *Taxus* trees.

## 4. Conclusions

Alternative sources for paclitaxel and its taxane derivatives constitute a distressing concern which led to the threat of extinction of *Taxus* genera trees as well as supply limitation due to the poor yield of the taxane purification methods. Endophytic fungi, particularly these belonging to the *Aspergillus* and *Trichoderma* genus and others identified in this publication, show great potential for taxane production, including paclitaxel. Further research is needed to optimize the fungus culture media and conditions in order to enhance the desired end product extraction; nevertheless, this study paves the way towards the development of a viable industrial production method for paclitaxel. Indeed, the microorganisms encountered in this research open up the possibility of using a mixed culture system that would produce the amounts of paclitaxel required for widespread cancer treatment with this compound. Further research is needed to identify the precise metabolic routes used by these microorganisms to synthetize taxanes and eventually clone and transfer these to microorganisms more adapted to mass culture, such as yeast.

## Figures and Tables

**Table 1:** Baccatin III production in different biological systems.

Strain	Detection Method	Baccatin III Production	Reference
*Diaporthe phaseolorum*	HPLC	Baccatin III content of full and drained PDB culture is 0.219 mg/L and 0.193 mg/L, respectively, and 0.014 mg/g of dry mass.	[26]
*Didymostilbe* sp.	LC-MS	8–15 µg/L	[27]
*Ozonium* sp.	HPLC-MS	12–18 µg/L	[28]
*Cladosporium, Alternaria, Fusarium*	HPLC	23.5–139.2 mg/kg dw	[29]
*F. solani*	Enzymatic Competitive inhibition immunoassay (CIEIA)	4.9–128.3 µg/L	[30]
Isolate TgO and *Aspergillus* sp.	LC–QTOF MS/MS	0.7–1.35 µg/L	This study

## Data Availability

The original contributions presented in this study are included in the article/Supplementary Materials. Further inquiries can be directed to the corresponding authors.

## Supplementary Materials

The following supporting information can be downloaded at: https://www.mdpi.com/article/10.3390/microorganisms13020300/s1, Figure S1: Absorbance curve; Figure S2: Molecular phylogenetic analysis by maximum likelihood method; Table S1: Table of paclitaxel and baccatin III concentration vs. superficies under the HPLC
