# Supplementary material for: Taxane-Producing Fungi Isolated from Taxus globosa Tree Bark
Source: Microorganisms. 2025 Jan 29;13(2):300. doi: 10.3390/microorganisms13020300 (PMC11858034; doi:10.3390/microorganisms13020300)
Supplement: Supplementary file 1 [file microorganisms-13-00300-s001.zip › microorganisms-3374362-supplementary.pdf]

Supplementary material

Table S1. Table of paclitaxel and baccatin III concentration vs superficies under the HPLC

ppm	Baccatin III	Paclitaxel (IS)
10	30.930	7.960
10	29.473	8.291
10	33.487	8.216
7.5	20.659	5.327
7.5	21.911	4.973
7.5	21.839	5.419
5	16.866	3.686
5	14.270	3.685
5	17.796	4.299
2.5	7.375	1.608
2.5	8.222	2.012
2.5	7.598	1.800
1	2.958	0.640
1	3.518	0.840
1	3.492	0.729
0.5	1.419	0.325
0.5	1.683	0.398
0.5	1.445	0.414
0.1	0.301	0.081
0.1	0.313	0.079
0.1	0.367	0.080
0.05	0.152	0.041
0.05	0.156	0.039
0.05	0.176	0.038
0.01	0.030	0.008
0.01	0.032	0.008
0.01	0.037	0.008

Figure S1. Absorbance curve

Paclitaxel

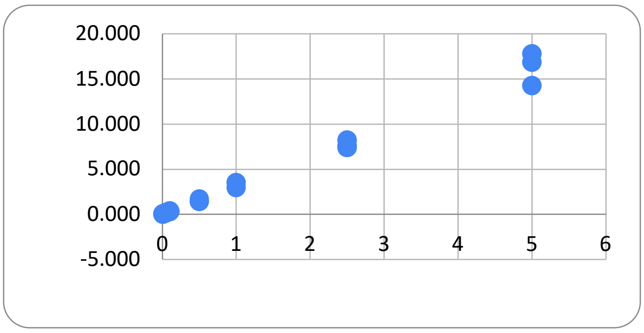

Baccatin III

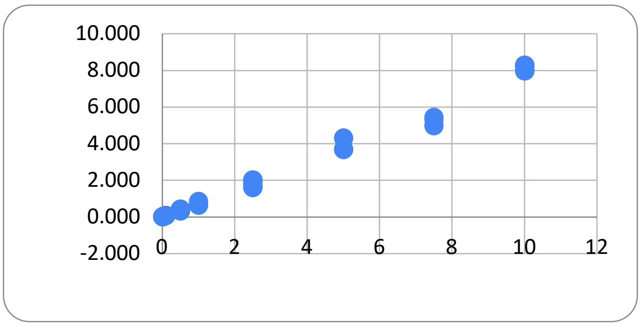

Figure S2.

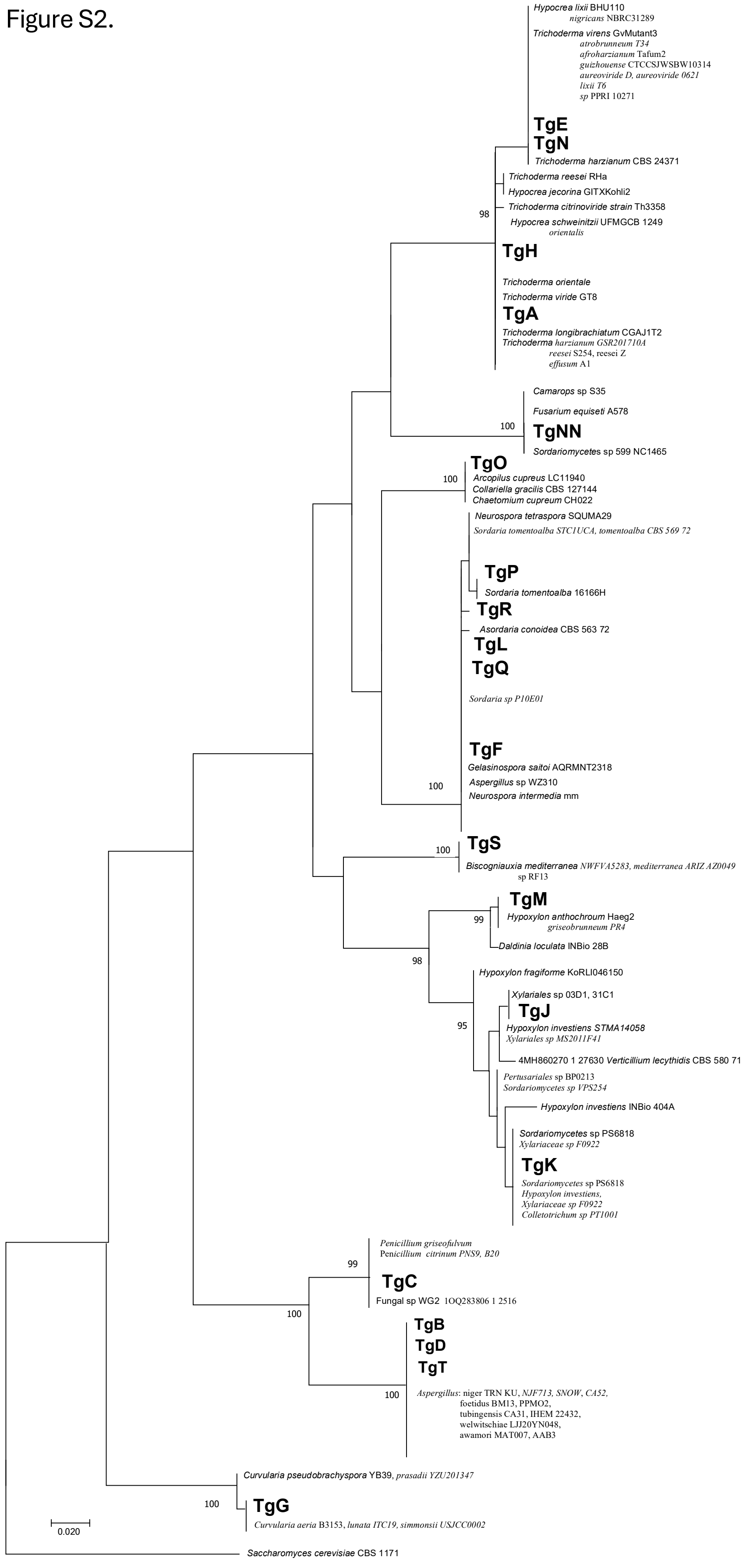

Figure S2. Molecular Phylogenetic analysis by Maximum Likelihood method
The evolutionary history was inferred by using the Maximum Likelihood method based on the Kimura 2-parameter model [1]. The tree with the highest log likelihood (-1539.54) is shown. The percentage of trees in which the associated taxa clustered together is shown next to the branches. Initial tree(s) for the heuristic search were obtained automatically by applying Neighbor-Join and BioNJ algorithms to a matrix of pairwise distances estimated using the Maximum Composite Likelihood (MCL) approach, and then selecting the topology with superior log likelihood value. The tree is drawn to scale, with branch lengths measured in the number of substitutions per site. The analysis involved 117 nucleotide sequences. All positions containing gaps and missing data were eliminated. There were a total of 245 positions in the final dataset. Evolutionary analyses were conducted in MEGA11.
